# Emerging Topical and Systemic JAK Inhibitors in Dermatology

**DOI:** 10.3389/fimmu.2019.02847

**Published:** 2019-12-03

**Authors:** Farzan Solimani, Katharina Meier, Kamran Ghoreschi

**Affiliations:** Department of Dermatology, Venereology and Allergology, Charité—Universitätsmedizin Berlin, Berlin, Germany

**Keywords:** inflammatory skin diseases, JAK (Janus kinase), JAK inhibition, pathophysiology, immunopathogenesis, autoimmune skin diseases, JAK/STAT pathway

## Abstract

Accumulating data on cellular and molecular pathways help to develop novel therapeutic strategies in skin inflammation and autoimmunity. Examples are psoriasis and atopic dermatitis, two clinically and immunologically well-defined disorders. Here, the elucidation of key pathogenic factors such as IL-17A/IL-23 on the one hand and IL-4/IL-13 on the other hand profoundly changed our therapeutic practice. The knowledge on intracellular pathways and governing factors is shifting our attention to new druggable molecules. Multiple cytokine receptors signal through Janus kinases (JAKs) and associated signal transducer and activators of transcription (STATs). Inhibition of JAKs can simultaneously block the function of multiple cytokines. Therefore, JAK inhibitors (JAKi) are emerging as a new class of drugs, which in dermatology can either be used systemically as oral drugs or locally in topical formulations. Inhibition of JAKs has been shown to be effective in various skin disorders. The first oral JAKi have been recently approved for the treatment of rheumatoid arthritis and psoriatic arthritis. Currently, multiple inhibitors of the JAK/STAT pathway are being investigated for skin diseases like alopecia areata, atopic dermatitis, dermatomyositis, graft-versus-host-disease, hidradenitis suppurativa, lichen planus, lupus erythematosus, psoriasis, and vitiligo. Here, we aim to discuss the immunological basis and current stage of development of JAKi in dermatology.

## Introduction

**Figure 1:**
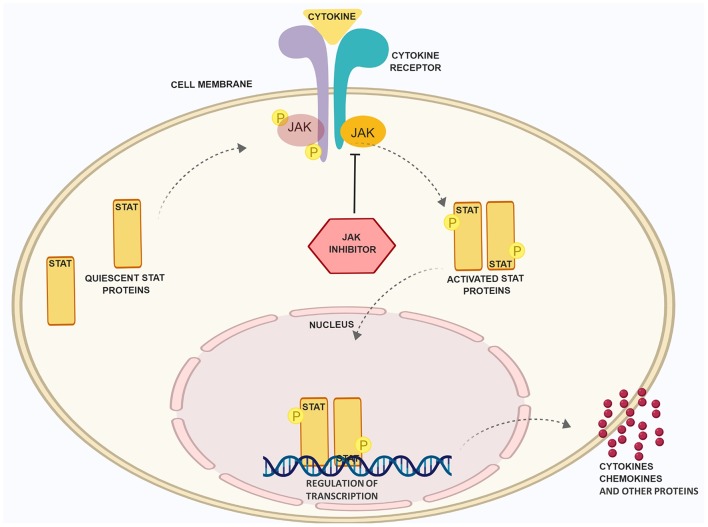
Schematic presentation of the JAK/STAT pathway and the role of JAK inhibitory drugs. Binding of cytokines to receptors, which rely on the JAK/STAT pathway for signal transduction, leads to phosphorylation of JAK and STAT proteins. The latter dimerize, translocate into the nucleus and regulate the expression of inflammatory factors. JAK inhibitors (JAKi) prevent JAK phsophorylation and STAT activation. Figure was created with the help of Biorender.com.

The classification of skin diseases and their treatment options are becoming more and more complex. While for a long period of time the morphology of diseased skin was prominent for disease classification and therapeutic procedures, we now have the methodologies for a deep analysis of molecular processes and immunological pattern analysis responsible for the pathophysiological alterations. These advances enlarged our therapeutic repertoire in dermatology remarkably. Deep analysis of skin biopsies allows us to define pathophysiological factors like cytokines, receptors or signaling molecules that are ultimately present at different levels in distinct skin diseases (1–3). This ultimately leads to the identification of new targets and, if these targets are druggable, to new treatments. One striking example of the aforementioned process is the development of a therapeutic variety with monoclonal antibodies (mabs) and small molecules in the treatment of psoriasis (PSO). Traditionally, PSO was treated with topical corticosteroids or dithranol, with phototherapy or immunosuppressive drugs such as cyclosporine. Nowadays, patients with this chronic inflammatory disease are frequently treated with biologicals, either of the first generation (anti-TNF) or the second generation (anti-IL-17/anti-IL-23) (4–7). In recent years, our better understanding of further inflammatory skin diseases such as lupus erythematosus (LE), lichen planus (LP) or atopic dermatitis (AD) also resulted in the development of novel treatment strategies (8–11). At the same time, we have gained more insight into the role of proteins propagating the intracellular effects of activated cytokine receptors (12). Again, when we pay attention to PSO, small molecules like dimethylfumarate or apremilast are good examples of intracellularly acting compounds that interfere directly or indirectly with signaling pathways (13). Most recently, first inhibitors of signaling proteins directly linked to cytokine receptors have been introduced into the clinics for treating patients with psoriatic arthritis (tofacitinib) and rheumatoid arthritis (tofacitinib and baricitinib). These inhibitors target so-called Janus kinases (JAKs), a family of four proteins: JAK1, JAK2, JAK3, and TYK2 (12). These proteins modulate the inflammatory process by activation of intracytoplasmic transcription factors called signal transducer and activator of transcription (STAT). Once activated, these proteins form dimers, translocate into the nucleus and either positively or negatively modulate the expression of thousands of different genes (12, 14) (Figure 1). Since JAK inhibition is not only restricted to systemic drugs administrated orally but has also been developed as a topical treatment option, it is not surprising that inhibitors of JAKs are receiving growing attention from dermatologists and are tested as systemic and/or topical treatment options in various skin diseases (15).

## The JAK Family and Their Function

**Figure 2:**
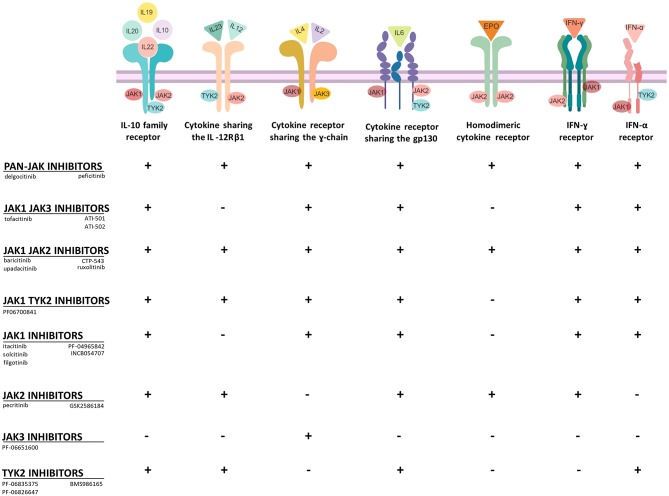
Selectivity of JAKi toward type I and type II cytokine receptors. JAKi display different capacities to block cytokine receptor signaling. Pan-JAK inhibitors for example have a broad inhibitory effect, while drugs, which target selectively JAK3 or TYK2 have a more limited mode of action (+, inhibition; -, no inhibition). Figure was created with the help of Biorender.com.

The importance of protein kinases and their crucial enzymatic activity was initially determined in 1966 by the work of Krebs and Fischer who showed the essential role of phosphorylation as a mechanism of cell physiology (16). The primary function of protein kinases is to transfer phosphate groups from adenosine triphosphate (ATP) or guanosine triphosphate (GTP) to the hydroxyl groups of amino acids of their protein targets (12). This mechanism is also important for cytokine receptors, which lack intrinsic enzymatic activity (Figure 2). In principle, the binding of cytokines to their receptors typically initiates an inflammatory signal (Figure 1). A large group of cytokines composed of central interleukins (IL) such as IL-2, IL-6, IL-12, IL-21, IL-22, IL-23, or interferons such as IFN-γ interacts with so-called type I and II cytokine receptors (Figure 2). Both of these receptor types lack intrinsic enzyme activity and strongly rely on JAKs for signal transduction (17). After binding, recruited JAKs initiate a signaling pathway from cellular membrane that ultimately should end in the nucleus: cytokine receptors of type I and II undergo oligomerization, leading to the recruitment of JAKs, which (auto-)phosphorylate tyrosine residues including such within the receptor chains (Figure 1). Successively, STAT proteins will be recruited, which then bind to the phosphorylated residues and become activated due to phosphorylation by JAKs. The now activated STAT proteins undergo dimerization. This last step enables the translocation of STAT proteins into the nucleus and the modulation of gene expression (14). Interestingly, most of cytokine receptors use a combination of JAKs for their activity; this could therefore hamper the idea of targeting single JAKs and instead favor a rationale for the use of pan-JAKi under certain settings. However, one should consider that toxicities may limit a strong and ubiquitous JAK blockade (Table 1).

**Table 1:** Selected adverse effects observed under treatment with JAK inhibitors.

**Frequent adverse events observed under treatment with JAK inhibitors**
Infections - Nasopharyngitis - Upper respiratory tract infections - Urinary tract infections
Herpes virus reactivation - Herpes Zoster - Lip/oral herpes simplex infections
Gastrointestinal disorders - Nausea - Diarrhea
Blood/serum changes
- Elevation of liver enzymes [aspartate aminotransferase (AST). alanine aminotransferase (ALT)] - Hyperlipidemia (increase in cholesterol, triglycerides) - Increase in bilirubine - Increase in creatine phosphokinase
Blood cell count alteration - Decrease in hemoglobin level - Decrease in white blood cell count - Neutropenia
**Rare adverse events reported under treatment with JAK inhibitors**
- Thromboembolic events - Non-melanoma skin cancers - Solid cancers

## JAK Deficiency in Humans and Mice

The crucial role of JAKs and the relevance of their function can be observed in patients with mutations in genes encoding JAKs or in mice carrying JAK mutations. Here, we will briefly summarize some loss of function (LOF) mutations of JAKs found in humans or by genetic manipulation of mice. Functional deletion of JAK1 or JAK2 in mice results in perinatal or embryonic lethality, respectively. Likewise, reports regarding individuals with deficiency of either JAK1 or JAK2 proteins have not been described, indicating that functional JAK1 and JAK2 are required for embryonic development and survival. In contrast, JAK3 knockout (KO) mice and TYK2 KO mice are both viable. JAK3 is widely expressed in immune cells. Consequently, JAK3 KO mice present a strong reduction of T and B cell numbers and residual immune cells show an impaired activity, exposing mice to multiple infections (18). In agreement with these findings, mutations within the JAK3 gene can present in humans as severe combined immunodeficiency syndrome (SCID) (19, 20). Patients with SCID lack T, NK and diverse B cell populations in the peripheral blood. As a result of this mutation, these patients are constantly exposed to a risk of bacterial, fungal or viral infections (21). The devastating effect on lymphocytes explains the crucial role of JAK3 in lymphocyte biology. JAK3 is the only JAK protein capable of phosphorylating receptors carrying the γc receptor and this receptor chain is exclusively used by receptors for IL-2, IL-4, IL-7, IL-9, IL-15, and IL-21 (20). LOF mutations within the TYK2 gene are even rarer than JAK3 mutations (22). The few patients reported carrying the mutation show an increased susceptibility for infections, like severe infections of the skin. TYK2 LOF mutations essentially block the signal transduction of the receptors for IL-12, IL-23, and type I IFN (IFN-α/β), resulting in impaired IFN-γ^+^ Th1 responses and possibly IL-17^+^ Th17 responses (23). Similarly, TYK2 KO mice present a defective response to aforementioned stimuli and have an impaired macrophage activity with increased risk for viral and bacterial infections (24). Based on the selective expression and function of JAK3 in the hematopoietic system, early research focused on the generation of selective JAK3 inhibitors. However, the first inhibitors brought to clinical practice were less selective than expected. The JAK3 inhibitor tofacitinib showed additional activities against JAK1 and to some extent toward JAK2. In contrast, baricitinib is a JAK1/JAK2 inhibitor. However, even these less selective JAKi seem to have acceptable toxicities. Yet, recent efforts in the field of pharmacological chemistry demonstrated that it is possible to generate JAKi with improved selectivity. For instance, second generation JAK3 inhibitors with high selectivity have been synthesized in the last years (25–28). Accumulating data from published studies and ongoing clinical trials will show which type of JAK blockade—low or highly selective compounds—will improve skin diseases without inducing major side effects.

## Rational for the Use of JAKi in Dermatology

The perpetuation of inflammation in diseased skin strongly relies on the interaction between cytokines, immune, and tissue cells propagating distinct inflammatory cascades. Some of these immunological processes in the skin, as in PSO or AD, have been deeply elucidated in recent years. The findings led to a radical change of therapeutic approaches. Based on our better understanding of the immunological mechanisms in PSO and AD, a variety of mabs targeting cytokines and small molecules interfering with intracellular signaling pathways have been developed. In this context, it is not surprising that JAKi are gaining increasing attention for the treatment of inflammatory skin diseases (15, 29, 30). Differently than biologics, which target cytokines by intravenous or subcutaneous injection, JAKi target cytokine signaling by either oral or topical administration. The latter way of application may minimize the risk of side effects as observed by systemic JAK inhibition (Table 1). Moreover, topical JAKi do not bear the risk of skin atrophy or telangiectasia as observed under long-term use of topical corticosteroids. In the following paragraphs, we aim to present the current position of JAKi in dermatology focusing on inflammatory skin diseases for which JAKi are at least in phase II investigation according to announced trials at clinicaltrials.gov.

## Alopecia Areata

**Figure 3:**
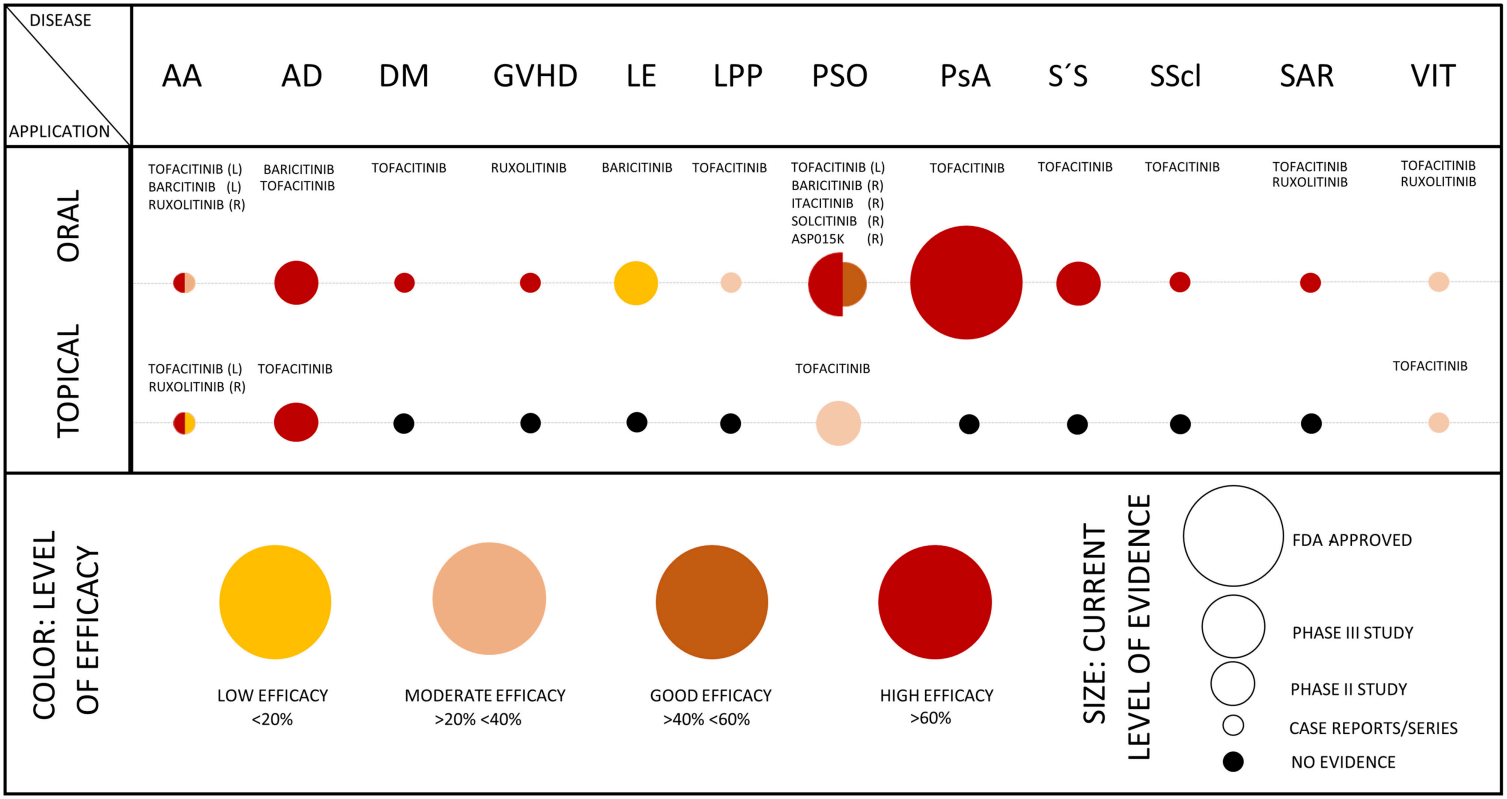
Efficacy of JAKi in dermatology. The scheme summarizes the level of efficacy (as represented by colors) and the level of evidence (as represented by size of circles) in the indicated skin diseases. In diseases, where results from phase II/III studies are available as published, evaluation of JAKi in case series or single case reports was omitted. The scheme was adapted from Eyerich et al. (1). AA, alopecia areata; AD, atopic dermatitis; DM, dermatomyositis; GVHD, graft versus host disease; LE, lupus erythematosus (efficacy on skin lesions); LPP, lichen planopilaris; PSO, psoriasis; PsA, psoriasis arthritis; S'S, Sjögren's syndrome; SScl, systemic sclerosis; SAR, sarcoidosis; VIT, vitiligo; (L), left; and (R), right half of the circle.

Alopecia areata (AA) is the most common immunological cause of hair loss (31). AA can affect both, adults and children. Although certain ethnic groups are more frequently affected than others, the disease does not prefer certain hairs types or color (31). Sudden hair loss is a hallmark of AA. In most of the cases, it appears in a circular well-circumscribed region of the scalp or the beard. AA can eventually lead to the loss of all the hairs of the scalp (alopecia totalis) or, in its most severe form, to the loss of hair of the whole body (alopecia universalis) (31). Frequently, the disease is accompanied by atopic dermatitis or by other autoimmune disorders such as autoimmune thyroiditis (32, 33). AA is determined by the loss of the immune privileged status of the hair follicles, which are then attacked by autoreactive CD8^+^ T cells and by NK T cells. Remarkably, hair follicles have developed different mechanisms to maintain their privileged immune status. For example, they express molecules such as transforming growth factor (TGF-)β1 and TGF-β2, α melanocyte stimulating hormone (α-MSH) and macrophage migration inhibitory factor (MIF), which hinder activation of T cell and NK T cell functions (31). Additionally, genetic background plays a prominent role in AA. Studies revealed that patients with positive family history for AA have a poorer prognosis and show a recalcitrant disease course often not responding to any treatments (34). Genome-wide-association studies (GWAS) showed the presence of diverse susceptibility loci possibly involved in the pathogenesis of AA like genes encoding for *HLA, ULB1, IL12RA*, and *PTPN22* (35). *ULB1* encodes for ligands involved in the activation of NKG2D cells, which in C3H/HeJ mice have been shown to be responsible for the destruction of hair follicles (36). Recent findings increased the evidence that JAKs play a crucial role in the pathogenesis of AA. Recipient mice of skin grafted C3H/HeJ mice were treated with mabs targeting IFN-γ, IL-2, and IL-15 each preventing the development of severe AA (36). Furthermore, Xing et al. showed that AA patients and experimental AA mouse models present increased levels of phosphorylated STAT proteins, specifically STAT1, STAT3, and STAT5. These STAT proteins are activated downstream the signals from IFN-γ, IL-2, and IL-15. When using the experimental model of C3H/HeJ mice, systemic treatment with the JAK1/JAK3i tofacitinib or with the JAK1/JAK2i baricitinib protected from hair loss and topical application of tofacitinib stimulated hair regrowth in C3H/HeJ mice (36, 37). In addition, three AA patients were treated orally with the JAK1/JAK2 inhibitor ruxolitinib. This therapeutic approach led to a decrease of CD8^+^NKG2D^+^ cells and a rapid amelioration of AA (36). Further, microRNAs that influence the expression of the *IL2RA* gene seem to be implicated in AA pathogenesis (38). Although the rationale for treating AA with JAKi is given, the clinical introduction of JAKi for the treatment of AA is still at an early stage (Figure 3) (39). Data from phase 2 and 3 studies are needed to clarify the clinical impact of JAKi in patients with AA (Table 2; Figure 3). Some first clinical experiences with JAKi for the treatment of AA have been published and seem to be promising (40–42). Treatment with oral ruxolitinib showed hair regrowth in 9 out of 12 treated patients without causing severe adverse events. JAK1/JAK2 inhibition by ruxolitinib reduced the expression of cytotoxic markers and IFN-γ expression in lesional skin (43). Similarly, Kennedy-Crispin et al. reported hair regrowth in a subset of patients with AA, AA totalis, or AA universalis treated with tofacitinib 5 mg twice daily. During this study, only low-grade infections were documented (Table 1). However, the positive effect on hair regrowth was lost after treatment discontinuation (NCT02197455 and NCT02312882) (44). A recently published case series also reports from the phenomenon of hair loss rebound in AA patients following discontinuation of tofacitinib (45). These first experiences were confirmed by subsequent studies in adults and children using oral or topical JAKi, respectively (46–49). Currently, various double-blind placebo-controlled phase II and III trials testing the efficacy and safety of oral and topical JAKi in AA are ongoing, underlining the growing interest toward these compounds (Table 2). One caveat of this promising approach in AA is the preliminary experience that the effect of oral JAKi seems to be timely restricted and hair loss has been reported to reappear upon cessation of pharmacological JAK inhibition in a substantial number of patients (50).

**Table 2:** Clinical trial program of JAKi in alopecia areata and subtypes according to clinicaltrials.gov.

**Disease** **subtype**	**Inhibitor**	**Target**	**Administration**	**Phase**	**Study number**
AA AU AT	Tofacitinib	JAK1 JAK3	Topical	Phase II	NCT02812342
	ATI-501	JAK1 JAK3	Oral	Phase II	NCT0359427
	ATI-502	JAK1 JAK3	Topical	Phase II	NCT03759340
AA AT	Ruxolitinib	JAK1 JAK2	Topical	Phase II	NCT02553330
	Baricitinib	JAK1 JAK2	Oral	Phase II/III	NCT03570749
	Tofacitinib	JAK1 JAK3	Oral	Phase IV	NCT03800979
	Tofacitinib	JAK1 JAK3	Oral	Phase II	NCT02299297
	Delgocitinib	PanJAK	Topical	Phase II	NCT02561585
	PF06651600	JAK3	Oral	Phase II	NCT02974868
	PF06700841	JAK1 TYK2			
	PF-06651600	JAK3	Oral	Phase II	NCT03732807
	CTP-543	JAK1 JAK2	Oral	Phase II	NCT03811912
	CTP-543	JAK1 JAK2	Oral	Phase II	NCT03137381
eAA	Delgocitinib	PanJAK	Topical	Phase II	NCT03325296
	ATI-502	JAK1 JAK3	Topical	Phase II	NCT03551821

*AA, alopecia areata; AU, alopecia universalis; AT, alopecia totalis; eAA, eyebrows alopecia areata*.

## Atopic Dermatitis

Atopic dermatitis (AD) is a common chronic disease of the skin, which severely impairs patient's quality of life. The prevalence of AD is higher in children and adolescents, but the disease can manifest at any age. Clinically, AD is characterized by the presence of pruriginous eczematous lesions, typically on flexural sites (8). In almost 80% of AD patients, skin integrity is altered due to LOF mutations within the gene encoding filaggrin (*FGN*). Impaired *FGN* expression promotes the loss of transepidermal water resulting in xerosis and eczema. The skin barrier dysregulation together with the “atopic” cytokine milieu increases the risk for skin superinfections with bacteria or viruses (51). Historically, AD is thought to be a Th2 dominated disease. Nonetheless, there is growing evidence that the immunological environment of AD is not solely defined by Th2 cells and related cytokines (IL-4, IL-5, IL-10, IL-13, and IL-31) but also by cytokines linked to other Th cell responses such as IFN-γ (Th1), IL17, or IL-22 (Th17) and IL-33 (an alarmin) (8, 52–56). Dupilumab, a mab against IL-4Rα has shown efficacy in a large number of patients and is the first approved biological for AD (57). Nonetheless, the cytokine microenvironment in AD seems to be complex and patients seem to present with different cytokine signatures at different stages. Several mabs are in phase 2/3 development, including mabs neutralizing IL-13 (tralokinumab and lebrikizumab), IL-31 (nemolizumab) (58–60), and IL-33 (etokimab) (61). The neutralization of other cytokines is being tested in patients with AD including secukinumab and ustekinumab, mabs against IL-17A and p40 subunit of IL-23/IL-12, respectively. Given the diversity of cytokines implicated in the inflammatory processes of AD, there is growing interest toward JAKi, which could interfere with the signaling of multiple cytokines simultaneously (62). Tofacitinib and baricitinib are so far the best-studied JAKi in AD (63). The JAK1/JAK3i tofacitinib abrogates IL-4 signaling and the differentiation of Th2 cells (64). Oral tofacitinib has been shown to be effective in patients with moderate to severe AD (65, 66) and topical tofacitinib is effective in mild forms of AD (67). The latter formulation is of special interest in topical AD treatment, which is mostly based on topical corticosteroids or calcineurin inhibitors (67). The JAK1/JAK2i baricitinib is also being tested in AD patients. Guttman-Yassky et al. showed that oral baricitinib at a dose of 2 or 4 mg strongly ameliorates AD, helping patients to spare long-lasting application of topical corticosteroids (68). Other compounds, like the JAK1i oclacitinib or the pan-JAKi JTE-052 showed efficacy when orally administrated in small-sized cohorts (69, 70). Currently, two different phase III studies are investigating the efficacy and safety of oral baricitinib as a monotherapy for AD. Other systemically applied JAKi under clinical investigation for AD include the JAK1 inhibitors upadacitinib (71) and PF-04965842. Topically applied JAKi tested for adult and pediatric AD include the pan-JAKi delgocitinib (LEO124249) (Table 3). Positive results, in absence of severe side effects when using topical JAKi, could enormously help young patients in which the application of topical corticosteroids and systemic immunosuppressive drugs are often precarious.

**Table 3:** JAKi tested for atopic dermatitis.

**Disease** **subtype**	**Inhibitor**	**Target**	**Administration**	**Phase**	**Study number**
AD	Baricitinib	JAK1 JAK2	Oral	Phase III	NCT03334396
	Baricitinib	JAK1 JAK2	Oral	Phase III	NCT03334422
	Upadacitinib	JAK1	Oral	Phase II	NCT02925117
	Upadacitinib	JAK1	Oral	Phase III	NCT03738397
	Upadacitinib	JAK1	Oral	Phase III	NCT03607422
	Ruxolitinib	JAK1 JAK2	Oral	Phase II	NCT03011892
	Ruxolitinib	JAK1 JAK2	Oral	Phase III	NCT03745651
	Delgocitinib	PanJAK	Topical	Phase I	NCT03826901
	Delgocitinib	PanJAK	Topical	Phase II	NCT03725722
	PF-04965842	JAK1 JAK2	Oral	Phase III	NCT03796676
	PF-04965842	JAK1	Oral	Phase III	NCT03422822
	PF-04965842	JAK1	Oral	Phase III	NCT03720470
	PF-04965842	JAK1	Oral	Phase III	NCT03627767
	PF-04965842	JAK1	Oral	Phase II	NCT02780167
	PF-04965842	JAK1	Oral	Phase III	NCT0334960
	PF-04965842	JAK1	Oral	Phase III	NCT03575871
pAD	Upadacitinib	JAK1	Oral	Phase I	NCT03646604

*AD, atopic dermatitis; pAD, pediatric atopic dermatitis. Clinical trial program according to clinicaltrials.gov*.

## Dermatomyositis

Dermatomyositis (DM) is an ab-mediated autoimmune disease, which affects skin and muscles with variating extent (72, 73). A complex auto-ab profile (74) is helpful for diagnosing DM and for estimating the risk for developing distant organ involvement like lung, larynx, gastrointestinal tract, or heart during the disease course. Adult DM is frequently associated with malignancies (75). The pathophysiology is not completely understood, nonetheless multiple studies showed a pivotal role played by IFN-γ producing Th1 cells in DM (72, 76, 77). The standard treatment regimen in DM includes high-dose corticosteroids, non-steroidal immunosuppressants like azathioprine and often requires additional intravenous immunoglobulins (IVIG) (73, 78). In addition, diagnosis and treatment of disease-associated cancer is important. More recently, different case reports and small case studies showed a positive outcome of recalcitrant chronic DM after treatment with either ruxolitinib or tofacitinib (79–82) (Figure 3). Remarkably, JAKi seem to have a positive effect on lung involvement in DM (79, 82–84), which often represents a high-risk-mortality complication. The efficacy of tofacitinib is currently under investigation in a small cohort of 10 patients with recalcitrant DM (NCT03002649) (Table 4). If JAKi can improve non-cancer associated DM with limited toxicities, this treatment option would be of help and spare long-term use of high dose corticosteroids.

**Table 4:** JAKi trial in patients with dermatomyositis.

**Disease** **type**	**Inhibitor**	**Target**	**Administration**	**Phase**	**Study number**
DM	Tofacitinib	JAK1 JAK3	Oral	Phase I	NCT03002649

*Clinical trial program according to clinicaltrials.gov. DM, dermatomyositis*.

## Graft-Versus-Host Disease

Graf-versus-Host Disease (GVHD) is a serious though common systemic reaction following hematopoietic stem cell transplantation or donor lymphocyte infusion (85). It is caused by donor T lymphocytes activated by host antigens. Different organs may be affected during GVHD and the skin is one of the most commonly targeted organs. Cutaneous GVHD presents in an acute and/or a chronic form. Acute (aGVHD) is clinically characterized by maculopapulous rash, disseminated erythematous skin areas, which can eventually converge in a generalized erythroderma (86). The chronic GVHD (cGVHD) shows a heterogeneous clinical presentation and can develop from the acute form. Lesions often present as LP-like or scleroderma-like lesions, nonetheless the disease can present differently and resemble PSO or keratosis pilaris (86). Immunologically, the aGVHD of the skin shows a T cell infiltration made up mostly by Th2 type cells. In contrast, in cGVHD the skin contains an infiltrate rich in type 1 and type 17 cells. In peripheral blood, cGVHD patients present lower numbers of Treg cells and the expression of their transcription factor FOXP3 is strongly reduced (87, 88). Glucocorticosteroids, both as topical and in oral form together with other immunosuppressants and phototherapy or extracorporeal photopheresis are the mainstay treatment for cGVHD (89). Given the important role of T cells and their associated cytokines, the application of JAKi may theoretically be of benefit for patients with GVHD. Experimental studies showed that pro-inflammatory mediators such as IL-6 and IFN-γ play a major role in the pathogenesis of GVHD (90). Furthermore, polymorphisms on genes encoding for IL-6 and IFN-γ have been associated with disease severity (91, 92). Accordingly, Choi et al. showed that IFN-γ receptor (IFN-γR)-deficient allogeneic Tconv reduced the risk for GVHD in mice (93). They tested the efficacy of ruxolitinib in wild type T cells in two murine MHC mismatched models, showing that inhibition of JAK proteins induce an effect similar to the genetic loss of IFN-γR *in vitro* and *in vivo* (93). Other experimental studies confirmed that JAKi like ruxolitinib (94) or tofacitinib improve or even prevent severe GVHD (95). The benefit of ruxolitinib in preclinical models was translated to humans and, also here, showed improvement of GVHD in six patients (96). After preliminary encouraging results regarding the effect of ruxolitinib in cGVHD in humans (97) (Figure 3), different studies are now investigating the possibly beneficial role of JAK inhibition in aGVHD and cGVHD. A multicenter, randomized phase 2 trial has been initiated, to test the efficacy of oral ruxolitinib in steroid-refractory aGVHD (98). The GRAVITAS-301 study (NCT03139604) is investigating the efficacy of itacitinib (a JAK1 inhibitor) in acute GVHD in a randomized, double-blind placebo-controlled phase 3 study. This compound as well as ruxolitinib and baricitinib are currently under investigation in cGVHD (Table 5). Most recently, itacitinib even entered two phase I studies for prophylactic use to prevent GVHD after cell stem transplantation (Table 5).

**Table 5:** Clinical trials using JAKi in Graft-versus-Host-Disease according to clinicaltrials.gov.

**Disease** **subtype**	**Inhibitor**	**Target**	**Administration**	**Phase**	**Study number**
Prophylaxis of GVHD	Itacitinib	JAK1	Oral	Phase I	NCT03320642
	Itacitinib	JAK1	Oral	Phase I	NCT03755414
aGVHD	Ruxolitinib	JAK1 JAK2	Oral	Phase II	NCT02396628
	Ruxolitinib	JAK1 JAK2	Oral	Phase III	NCT02913261
	Ruxolitinib	JAK1 JAK2	Oral	Phase II	NCT03702698
	Ruxolitinib	JAK1 JAK2	Oral	Phase II	NCT03491215
	Ruxolitinib	JAK1 JAK2	Oral	Phase II	NCT02953678
	Itacitinib	JAK1	Oral	Phase I	NCT03497273
	Itacitinib	JAK1	Oral	Phase I/II	NCT03721965
	Itacitinib	JAK1	Oral	Phase III	NCT03139604
	Itacitinib	JAK1	Oral	Phase II	NCT03846479
	Pacritinib	JAK2	Oral	Phase I	NCT02891603
aGVHD cGVHD	Ruxolitinb	JAK1 JAK2	Oral	Phase II	NCT02997280
cGVHD	Baricitinib	JAK1 JAK2	Oral	Phase I/II	NCT02759731
	Itacitinib	JAK1	Oral	Phase III	NCT03584516
	Ruxolitinib	JAK1 JAK2	Oral	Phase II	NCT03616184
	Ruxolitinib	JAK1 JAK2	Oral	n/a	NCT03147742
	Ruxolitinib	JAK1 JAK2	Oral	Phase III	NCT03112603
	Ruxolitinib	JAK1 JAK2	Topical	Phase II	NCT03395340

*aGVHD, acute Graft-versus-Host-Disease; cGVHD, chronic graft-versus-host-diseases*.

## Hidradenitis Suppurativa

Hidradenitis suppurativa (HS) (also designated as acne inversa) is a chronic debilitating inflammatory skin disease frequently occurring in skin areas with substantial presence of follicles (99). The disorder is characterized by occlusion of follicular ducts with consequent increase in local bacteria. This process evolves in cyst rupture and generalized local tissue inflammation (99). Of note, it is probable that a sublatent skin inflammatory process precedes bacterial accumulation; this could be triggered e.g., by behavioral factors like smoking and obesity, which typically worsen the course of HS (99). Moreover, HS patients often carry mutations in the γ-secretase encoding gene *PSEN* (100). Due to the important role played by bacteria in HS, first line treatments are based on antibiotic therapy with anti-inflammatory properties (101, 102). Nonetheless, recent studies elucidated the role of cytokines present in the inflammatory milieu of HS skin showing an overexpression of IL-17A, IL-26, IFN-γ, IL-27, and IL-β, and, a concomitant downregulation of IL-22 (103, 104). This moved the focus of researchers from the role of bacteria to the function of cytokines in HS. Adalimumab, a mab against TNF, is the first biological approved for the treatment of HS (105). Targeting cytokines like IL-1 or IL-17/IL-23 have been reported to have mixed results (37, 106) or are presently under intensive clinical investigation. Given that cytokines are crucial in HS pathogenesis, inhibition of the JAK/STAT pathway could help to regulate the expression of inflammatory factors like IL-6 or IL-23 simultaneously. Clinical studies like NCT03607787 and NCT03569371 are evaluating the safety of INCB054707, a JAK1 inhibitor in HS (Table 6).

**Table 6:** Trials on JAKI in hidradenitis suppurativa according to clinicaltrials.gov.

**Disease** **type**	**Inhibitor**	**Target**	**Administration**	**Phase**	**Study number**
HS	INCB054707	JAK1	Oral	Phase II	NCT03607487
	INCB054707	JAK1	Oral	Phase II	NCT03569371

*HS, hidradenitis suppurativa*.

## Lichen Planus

LP is a common inflammatory disease of the skin and mucous membranes, clinically characterized by polygonal papules presenting a characteristic white, reticulate on their surface (Wickham striae). Frequently, the oral cavity is affected by LP (OLP) (107). Rarely, LP can affect the scalp, LP planopilaris (LPP), or the nails (nail LP) (107). A classic feature of this inflammatory disorder is the histological presence of a dense T cellular infiltrate disposed in a band like pattern (lichenoid infiltrate) (10). The need of new therapeutic options is given, since LP often presents a challenging chronic recalcitrant course (especially OLP) and the traditional therapeutic options are of limited efficacy. Treatment of LP is mainly based on oral or topical glucocorticosteroids, phototherapy, retinoids or immunosuppressive drugs such as cyclosporine or methotrexate (107). There is growing evidence that LP is a Th1 driven disease (10, 108, 109), although Th17-associated cytokines have also been reported in LP (110–112). A recent study showed that the T cell infiltrate presents high numbers of Th1/Tbet^+^ cells and the presence of IL-17A^+^ cells, disposed beneath the basal cells of the epidermis (10). Peripheral Th1/Th17 cell reactive against skin autoantigens further underlines the proposed autoreactive pathophysiology of LP (10). Finally, therapeutic blockade of IL-17 or of IL-23 seems to be a promising approach for the treatment of LP (11). The dominance of IFN-γ in LP skin suggests that JAK inhibition could be a therapeutic option. A small retrospective study consisting of 10 patients with LPP treated with oral tofacitinib as monotherapy or as adjunctive therapy showed mixed clinical efficacy in 8/10 patients (113). According to clinicaltrials.gov, the study NCT03697460 is testing the efficacy of ruxolitinib cream in LP (Table 7). It is likely, that JAKi will be a therapeutic option for patients with steroid-refractory LP variants. Given the beneficial effect of tofacitinib on nail PSO (114), a possibly similar effect on nail LP is conceivable. This should be addressed in the future, since nail LP is very painful and cicatrial.

**Table 7:** Trial on JAKi in lichen planus.

**Disease** **type**	**Inhibitor**	**Target**	**Administration**	**Phase**	**Study number**
cLP	Ruxolitinib	JAK1 JAK2	Topical	Phase II	NCT03697460

*Clinical trial program according to clinicaltrials.gov. cLP, cutaneous lichen planus*.

## Lupus Erythematosus

Lupus erythematosus (LE) is a severe, chronic autoimmune ab-mediated disorder with variable clinical presentation and difficult-to-treat clinical course. The disease is widely known for the presence of a butterfly-like rash on the face of affected patients with systemic LE (SLE) (115). Based on the diverse subtypes, LE can be restricted to the organ skin or present a systemic course with manifestation at multiple organs (115). Both environmental and behavioral factors (such as smoking or sun exposure) and genetic factors are meant to play a role in the pathogenesis of LE (115–117). Possibly, gut pathobionts and in general, bacterial and viral stimuli could trigger the onset of the disease (118). In LE there is evidence that multiple cytokines such as IL-16, IL-17, IL-18, and TNF are implicated in the inflammatory process. In any event, type I IFNs are thought to play a major role in LE pathogenesis (119). The latter family of cytokines strongly relies on the JAK/STAT pathway for signal transmission (Figure 2). In addition, the role of STAT proteins has been widely investigated (120). The pivotal role of STAT1 in LE has been analyzed in mice and in humans. In the MRL/lpr mouse model, STAT1 is overexpressed in both B and T cells. STAT1 gene silencing (STAT1^−/−^) in this mouse model is accompanied by a decrease of CD4^+^ producing IFN-γ T cells, a milder course of nephritis and a remarkable decrease of auto-ab levels (121–123). In humans diagnosed with LE as in mice, STAT1 is overexpressed in T and B cells. STAT1 levels correlate with clinical disease activity and with the level of expression of IFN-γ-induced genes (124–126). In addition, STAT3 and STAT4 seem to be relevant in the pathogenesis of LE. The lack of STAT3 seems to be protective in a LE murine model (127, 128). In MRL/lpr LE prone mice treatment with tofacitinib led to a decrease of auto-ab production and amelioration of nephritis and skin inflammation. Similarly, two different LE mouse models showed a decrease of several cytokines (IL-12, IL-17A, IFN-γ, and TNF), and a decrease of antinuclear auto-ab levels upon treatment with CEP-33779, a JAK2 inhibitor (129). However, in humans, the JAK1 inhibitor GSK2586184 showed to be ineffective in a small size study of patients with SLE (130). Conversely, baricitinib has been tried in patients with SLE in a double blind, randomized, placebo-controlled phase 2 trial (NCT02708095) (Figure 3) (131). Baricitinib was administered at 2 or 4 mg daily. Results showed that the 4 mg dose leads to a consistent clinical improvement in SLE, especially on arthritis. Of note, adverse events were almost comparable to the placebo group (132). At the time of this writing, different compounds targeting different JAKs are under clinical investigation for the treatment of SLE or discoid LE (Table 8).

**Table 8:** Clinical trials of JAKi in systemic and/or discoid lupus erythematosus according to clinicaltrials.gov.

**Disease** **subtype**	**Inhibitor**	**Target**	**Administration**	**Phase**	**Study number**
SLE	Tofacitinib	JAK1 JAK3	Oral	Phase I	NCT02535689
	Baricitinib	JAK1 JAK2	Oral	Phase III	NCT03843125
	Baricitinib	JAK1 JAK2	Oral	Phase III	NCT03616912
	GSK2586184	JAK1	Oral	Phase II	NCT01777256
	PF06835375	TYK2	Oral	Phase I	NCT03334851
	PF06700841	JAK1 TYK1	Oral	Phase II	NCT03845517
	BMS986165	TYK2	Oral	Phase II	NCT03252587
SLE/DLE	Tofacitinib	JAK1 JAK3	Oral	Phase 1	NCT03159936

*SLE, systemic lupus erythematosus; DLE, discoid lupus erythematosus*.

## Psoriasis Vulgaris

PSO is a common inflammatory skin disease with well-defined pathogenesis. Based on the research of recent years numerous targeted treatments have been developed for PSO (4). In its classic appearance, this chronic inflammatory disorder presents with erythematous scaly plaques, which are preferentially disposed at extensor sites and in areas of mechanic stress. In addition, scalp, nails and inverse regions are frequently affected. In around 20–30% of cases, patients with PSO also suffer from joint involvement (psoriatic arthritis, PSA) (4). There is a wide range of treatment possibilities for PSO. Topical treatments include glucocorticosteroids, vitamin D derivatives, and dithranol. While phototherapy is becoming less important, oral drugs like methotrexate, acitretin, dimethylfumarate, or apremilast are widely used (4). Cyclosporine should be avoided due to its toxicities compared to the numerous less-toxic alternatives. Besides oral drugs, various biologics have been established for PSO (6). The first generation of antipsoriatic biologics targets TNF. Since PSO is widely accepted as the prototypic Th17 driven disease, multiple second-generation biologics have been approved in the meanwhile. These either neutralize IL-17A, bind its receptor or target IL-23. Although the neutralization of IL-17A is effective in most patients, some patients do not respond. The implication of multiple cytokines like IL-6, IL-22, IL-23, or IFN-γ in psoriasis pathogenesis suggests that the inhibition of JAKs could be a powerful and more profound treatment than a treatment with a single mab. Of note, STAT3 is a key factor in IL-23/Th17 signaling. Active JAKs and active STAT3 are typically found in psoriatic skin (133, 134). The role of IL-23 and Th17 cells has also been studied in the imiquimod (IMQ) PSO mouse model. In this model, silencing of TYK2 by gene targeting abrogated skin inflammation (135). Likewise, the use of JAKi successively ameliorated skin inflammation with a concomitant decrease of cytokine levels in the IMQ PSO mouse model (136). In humans, Krueger et al. showed in a small cohort (*n* = 12) that treatment with tofacitinib (10 mg twice daily) ameliorated PSO and this was accompanied at a molecular level by the decrease of phosphorylated STAT1 and STAT3. Similarly, tofacitinib decreased epidermal thickness, reduced the number of T cells infiltrating the skin and suppressed the IL-23/Th17 pathway (137). These preliminary findings paved the way for JAKi application in humans with PSO. A phase III randomized double-blind placebo-controlled study (NCT01815424) demonstrated the efficacy of tofacitinib at doses of 5 or 10 mg twice daily in patients with moderate to severe PSO (138). The oral PSO trial (OPT) pivotal I and II studies confirmed these positive results by oral tofacitinib in chronic plaque PSO (Figure 3). Importantly, only 6% of treated patients experienced adverse events (139). Notably, treatment discontinuation was associated with a risk of relapse; however, re-initiation of the treatment rapidly resolved psoriatic inflammation (140). Another phase III randomized multicenter study showed that the efficacy of tofacitinib 10 mg twice daily is similar to the efficacy of etanercept 50 mg twice weekly in PSO (141). Tofacitinib (5 or 10 mg daily) is also beneficial in nail PSO (114). The OPAL study showed that the JAK1/JAK3i is highly effective in the control of PSA in patients not responding to anti TNF-α treatment. In 2018, tofacitinib 5 mg twice daily was approved by the FDA for the treatment of PSA (142). Under this therapeutic regimen tofacitinib seems to have an acceptable safety profile without severe adverse events even during long-term application (143, 144). Based on the experience with tofacitinib, numerous JAKi are tested as oral drugs or as topical formulation for PSO (Table 9) (145–147). So far, the efficacy of topical JAKi for psoriasis is not convincing (148–150). In a double-blind study, topical application of the JAK1/JAK2i ruxolitinib (1 or 1.5%) showed comparable reduction of skin inflammation as calcipotriene 0.005% cream or betamethasone dipropionate 0.05% cream (151). Oral JAKi are more promising. In a randomized, double blind, placebo controlled phase IIb study the JAK1/JAK2i baricitinib at 2, 4, 8, or 10 mg daily dose showed encouraging results in the treatment of moderate to severe PSO (152). Lastly, selective inhibition of TYK2 with the oral compound BMS-986165 seems to be another very promising therapeutic option for the treatment of PSO. In a phase II trial BMS-986165 given at 3, 6, 9, or 12 mg daily over a period of 12 weeks resulted in an impressive clearing of PSO compared to placebo (153). According to clinicaltrials.gov, the number of registered studies on JAKi for PSO is rapidly growing (Table 9). Both oral and topical compounds are currently under clinical investigation for PSO. These include the oral compounds solcitinib, filgotinib, upadacitinib, and delgocitinib (LEO124249), a topical pan-JAKi (154). The selective TYK2 inhibitors PF-06826647 and BMS-986165 are under investigation for PSO or PSO/PSA, respectively (Table 9). The expected results from these clinical trials will be a major step toward extending the therapeutic spectrum of PSO and PSA by oral compounds.

**Table 9:** JAKi trials in psoriasis vulgaris, psoriatic arthritis and psoriasis inversa according to clinicaltrials.gov.

**Disease** **subtype**	**Inhibitor**	**Target**	**Administration**	**Phase**	**Study number**
PSO	Ruxolitinib	JAK1 JAK2	Topical	Phase II	NCT00617994
	Peficitinib	PanJAK	Oral	Phase II	NCT01096862
	PF-06826647	TYK2	Oral	Phase II	NCT03895372
	PF-06826647	TYK2	Oral	Phase I	NCT03210961
	PF-06700841	JAK1 TYK2	Topical	Phase II	NCT03850483
	PF-06763809	n/a	Topical	Phase I	NCT03469336
	BMS-986165	TYK2	Oral	Phase III	NCT03624127
	BMS-986165	TYK2	Oral	Phase III	NCT03611751
PSA	Itacitinib	JAK1	Oral	Phase II	NCT01634087
	Upadacitinib	JAK1	Oral	Phase III	NCT03104374
	Upadacitinib	JAK1	Oral	Phase III	NCT03104400
	Tofacitinib	JAK1 JAK3	Oral	Phase III	NCT03736161
	Tofacitinib	JAK1 JAK3	Oral	Phase III	NCT03486457
	Tofacitinib	JAK1 JAK3	Oral	Phase III	NCT01976364
	Filgotinib	JAK1	Oral	Phase II	NCT03320876
	BMS-986165	TYK2	Oral	Phase II	NCT03881059
iPSO	Delgocitinib	PanJAK	Oral	Phase II	NCT02695940

*PSO, psoriasis vulgaris; PSA, psoriasis arthritis; iPSO, psoriasis inversa*.

## Vitiligo

Vitiligo is a skin disease with severe psychological impact on patients. The disease is characterized by the presence of white depigmented skin spots due to melanocytic destruction by self-reactive CD8^+^ T cells (155). There is an enormous need for effective treatment options since presently the limited treatment modalities are only effective in some patients. Currently, patients with vitiligo are either treated with topical glucocorticosteroids, topical calcineurin inhibitors (off-label), or with phototherapy (narrowband UVB). In addition, systemic administration of glucocorticosteroids or other immunosuppressive drugs are used. Mechanistically, type I immune responses seem to be responsible for the development of vitiligo (156–159). In lesional skin, overexpression of IFN-γ, which translates its intracellular signal through STAT1, and associated chemokines like CXCL10 and its receptor CXCR3 are found (158). The biological relevance of these clinical findings was confirmed in mouse models of vitiligo. For instance, transfusion of CXCR3^−/−^ PMEL T cells in vitiligo prone mice did not evolve to phenotypic alterations. Similarly, interfering with the CXCL10-CXCR3 interaction through targeting of CXCL10 with mabs results in vitiligo reversal in mice with established disease (160). These and other findings helped to elucidate the copious immunological players dominating vitiligo and underlined the crucial role of the JAK/STAT pathway and their related factors in the pathogenesis of this disorder. First small-size case series report from the efficacy of JAKi in vitiligo (161). Rothstein et al. used ruxolitinib 1.5% cream over a period of 20 weeks in a small group of 12 patients with vitiligo. They reported successful repigmentation of facial spots, while repigmentation of other anatomical sites showed mixed results (162). Similar results with better responses of facial spots than non-facial spots were reported by a recently published cohort study with 16 patients receiving 2% tofacitinib cream (163). A retrospective case study of 10 patients treated with oral tofacitinib 5 or 10 mg once daily suggests that a combination of phototherapy with JAKi is therapeutically more effective than JAKi monotherapy (164) (Figure 3). These reports indicate that JAKi in combination with UV exposure are required for stimulating repigmentation. Currently, different JAKi are under investigation in phase II trials for topical application in patients with vitiligo (Table 10).

**Table 10:** Clinical trials on JAKi for treating patients with vitiligo according to clinicaltrials.gov.

**Disease** **type**	**Inhibitor**	**Target**	**Administration**	**Phase**	**Study number**
Vitiligo	Ruxolitinib	JAK1 JAK2	Topical	Phase II	NCT03099304
	Ruxolitinib	JAK1 JAK2	Topical	Phase II	NCT02809976
	ATI-502	JAK1 JAK3	Topical	Phase II	NCT03468855
	PF-06651600 PF-06700841	JAK3 JAK1 TYK2	Oral	Phase II	NCT03715829

## Possible Future Applications

The introduction of JAKi is enlarging the therapeutic repertoire of dermatologists and is proving efficacy in numerous inflammatory diseases. Disorders like vitiligo or AA are in high need for efficient treatments. Increasing evidence confirming the beneficial role of systemic or topical JAKi in the treatment of these disorders is accumulating. Since the JAK/STAT signaling pathway plays a crucial role for many cytokines (Figure 2), a variety of inflammatory dermatological disorders may benefit from this new class of immunomodulators. For instance, lichen sclerosus et atrophicans (LSA) is an inflammatory disorder of skin and genital mucosa associated with severe pruritus and scaring. Many patients do not respond adequately to topical steroids (165). Due to its frequent recalcitrant course, there is an urgent need of new therapeutics for LSA. Inflammatory factors like IFN-γ, CXCL10, and CCR5 are highly expressed in lesional skin, suggesting that LSA is a Th1-dominated disorder (166). Oral or topical application of JAKi could stop the inflammatory circuit in LSA. Case reports on positive effects of JAKi exist for LSA and for patients with other sclerosing skin diseases like morphea, eosinophilic fasciitis, or systemic sclerosis (167, 168). These results are in agreement with experimental findings demonstrating a pivotal role of STAT3 in the activation of profibrotic pathways *in vivo* and *in vitro* (169). JAK inhibitors also improve inflammatory bowel diseases (IBD) like ulcerative colitis. Based on this data, it can be argued that IBD-associated skin diseases such as cheilitis granulomatosa (CG), pyoderma gangrenosum (PG) or erythema nodosum (EN) could also benefit from JAKi. CG shows a similar immunological profile as Crohn's disease with overexpression of IFN-γ (170–172). Patients with CG benefit from drugs such as thalidomide or lenalidomide (173, 174), which suppress Th1 cell responses (175). In contrast, analysis of PG and EN lesional skin revealed overexpression of TNF and STAT3, the latter one suggesting a rationale for the application of JAKi in these diseases (176). Other inflammatory diseases, where the use of JAKi seems to be desirable include sarcoidosis (177–180), and Sjögren's syndrome (SS), autoimmune diseases with strong molecular association to the JAK/STAT signaling pathway (181–184). Patients with blistering skin diseases like bullous pemphigoid, dermatitis herpetiformis Duhring and pemphigus vulgaris may also benefit from JAKi (185–187). JAKi have been shown to inhibit auto-ab production in preclinical models (188). Moreover, different studies highlighted the crucial role of STAT1 and STAT3 in transporting the signals derived from IL-6 and IL-21, two crucial cytokines for germinal centers formation. Lack of IL-6 and IL-21 leads to a strong reduction of T follicular helper (T_FH_) cell levels and consequently to impaired germinal center formation and suppression of IgG levels, since T_FH_ cells provide unavoidable help for B cells' ab production (189, 190). Due to this preliminary data, JAKi could dramatically improve patients' quality of life in auto-ab-mediated bullous skin disorders, where high doses of oral glucocorticosteroids are currently the therapeutic gold standard and where the need of new therapeutic approaches is high, even though the B cell depleting mAb rituximab has been approved recently (191, 192). Another group of skin disorders that could benefit from JAKi are eczema. Delgocitinib, a pan-JAKi is currently under investigation in patients with chronic hand eczema (Table 11) and shows some first promising results (193). In nickel-induced allergic contact dermatitis there is evidence of an important involvement of the JAK/STAT pathway (194). Accordingly, mouse models for contact dermatitis show positive response to treatment with topical tofacitinib (JAK1/JAK3i) (195, 196), thus suggesting a possible application of topical JAKi also in humans. In Behcet's disease, an autoimmune disorder with unsolved pathogenesis experimental studies showed overexpression of Th17 related genes, overexpression of type I IFN-inducible genes and activation of the JAK/STAT pathway (197–199). These first experimental results pave the way for a future tentative application of JAKi in this disorder. Lastly, JAKi could improve autoinflammatory disorders. A patient with synovitis, acne, pustulosis, hyperostosis, and osteitis (SAPHO), has been successfully treated by a combination of methotrexate and oral tofacitinib (200). Similarly, the clinical trials NCT01724580 and NCT02974595 proved efficacy of baricitinib in a small group of patients affected by CANDLE (chronic atypical neutrophilic dermatosis with lipodystrophy and elevated temperatures) and SAVI (stimulator of IFN-genes associated vasculopaty with onset in infancy) (201, 202).

**Table 11:** Clinical trials of JAKi in chronic hand eczema. cHE, chronic hand eczema according to clinicaltrials.gov.

**Disease** **type**	**Inhibitor**	**Target**	**Administration**	**Phase**	**Study number**
cHE	Delgocitinib	PanJAK	Topical	Phase II	NCT03683719
	Delgocitinib	PanJAK	Topical	Phase II	NCT02664805

## Concluding Remarks

The introduction of JAKi in dermatology will revolutionize the therapeutic outcome of various inflammatory skin diseases. There is growing evidence that the JAK/STAT signaling pathway is a key pathway in numerous skin disorders. However, we are facing several hurdles. First, the development of selective JAKi was harder than initially thought. First generation JAKi, like tofacitinib and baricitinib, which were thought to be highly selective, target more than one single JAK. This is not necessarily a disadvantage regarding efficacy, but of course may bear a higher risk for toxicities (17). For instance, significant inhibition toward JAK2 leads to anemia. Some unexpected adverse events were also observed when using first generation JAKi (203–205). One example is the increase in cholesterol levels by tofacitinib (144, 206–208), which could be a paradox effect induced by the reduction of inflammation (209). The more recently developed JAKi seem to have improved selectivity (25, 27, 210). The other caveat for treating skin disorders via topical route is to generate formulations that allow small molecules like JAKi to penetrate the skin. Here, improvements like nanocarriers may be helpful (211–213). We still have a high therapeutic need for several skin diseases like vitiligo, AA, AD, LPP, LSA, and others. JAKi as topical formulation or systemic drug could be an outstanding improvement without side effects typically observed when using topical or systemic glucocorticosteroids. Yet, in the majority of the aforementioned skin diseases, we urgently need more evidence and larger double-blind placebo-controlled studies to confirm efficacy and safety of JAKi. The safety profile of JAKi reported so far seems to be acceptable, at least when used as monotherapy. One important question, which could favor the introduction of JAKi in dermatological daily routine, is the comparison of these with the well-established application of mabs. Even though studies directly comparing JAKi with biologicals are widely missing for dermatological indications, the anti-inflammatory properties of JAKi are conceivably profound. Nonetheless, the safety profile of these new compounds compared to the safety profile of mabs seem not to be very different (214–216). Experience from the here discussed studies show that severe adverse events are rare. Common adverse events like infections of the upper respiratory tract or common disturbances such as headache or diarrhea can be frequent, but easy to manage (217–219) (Table 1; Supplementary Tables 1–3). The reactivation of herpes zoster infection seems to be more frequent in patients treated with JAKi compared to those receiving biologicals and therefore vaccination prior to treatment initiation should be discussed (220–223). Blood count alterations seem to be reversible and normalize after withdrawal (224, 225) (Table 1). Published studies with different JAKi indicate an increased risk for bacterial and viral infections (226–230). From an economic point of view the lower production costs of JAKi compared to mabs may favor their application in dermatology (231). However, the simultaneous inhibition of multiple cytokines by systemic JAKi bears the risk for fatal outcomes during severe infections and possibly also the risk for cancer development on the long run (232). Moreover, the dose and half-life of JAKi is an important issue. It will be crucial to lower the threshold of JAK/STAT activation but not to permanently block this pathway. Notably, another major issue which must be addressed in the future and which is presently unknown is the impact of these drugs on long-term treatments setting. First data from non-dermatological studies will be available soon, but data from longer observational periods after approval for dermatological indications are needed (233–236). This will be important for calculating the risk for both, infectious and tumor diseases (237). In conclusion, JAK inhibitory drugs are emerging as a new and effective treatment for various diseases. So far, the results arouse enthusiasm among dermatologist; nonetheless, further studies will decide about the real-world efficacy and safety profiles of this new class of immunomodulators.

## Author Contributions

FS and KG designed the manuscript and designed figures and tables. FS, KM, and KG drafted the manuscript and approved the final version of the manuscript. KG revised critically the final version of the manuscript.

### Conflict of Interest

KG has received honoraria or travel expenses for lecture and research activities from Abbvie, Almirall, Biogen, Boehringer Ingelheim, Bristol-Myers Squibb, Celgene, Delenex, Eli Lilly, Galderma, Janssen-Cilag, Medac, MSD, Novartis, Pfizer, and UCB Pharma. KM has received honoraria or travel expenses for lecture and research activities from Abbvie, Celgene, Janssen-Cilag, and UCB Pharma. The remaining author declares that the research was conducted in the absence of any commercial or financial relationships that could be construed as a potential conflict of interest.
