# Supplementary material for: Emerging Topical and Systemic JAK Inhibitors in Dermatology
Source: Front Immunol. 2019 Dec 3;10:2847. doi: 10.3389/fimmu.2019.02847 (PMC6901833; doi:10.3389/fimmu.2019.02847)
Supplement: Supplementary file 1 [file Table_1.DOCX]

**Supplementary Table 1**: Selected adverse events in phase II clinical trials for atopic dermatitis

| **Disease type** | Atopic Dermatitis | | | | | |
| --- | --- | --- | --- | --- | --- | --- |
| **Reference** | Bissonnette et al.  2016 | | Guttmann-Yaski et al. 2019 | | | |
| **Study Type** | Phase IIa  (NCT02001181) | | Phase II  (NCT02576938) | | | |
| **Drug** | 2% Tofacitinib  Ointment BID | Vehicle BID | Baricitinib 2mg | Baricitinib 4mg | | Placebo |
| **N^○^ of**  **patients** | 35 | 34 | 37 | 38 | | 49 |
| **Observational period** | 4 weeks | | 16 weeks | | | |
| **Infections of the upper respiratory tract** | 1 (2.9) | 1 (2.9) | 1 (3) | 2 (5) | 1 (2) | |
| **Nasopharyngitis** | 2 (5.7) | 2 (5.9) | 1 (3) | 3 (8) | 1 (2) | |
| **Other infections** | 3 (8.7) | 0 | 1 (3) | 2(6) | 5 (10) | |
| **Fungal**  **infections** | 0 | 0 | 0 | 1 (3) | 0 | |
| **Skin infections** | 1 (2.9) | 0 | 3 (9) | 4(12) | 3 (6) | |
| **Herpes virus**  **reactivation** | n/a | n/a | 0 | 1 (3) | 1 (3) | |
| **Liver / renal paramater increase** | 0 | 5 (14.6) | 1 (3) | 5(13) | 0 | |
| **Blood Count alteration** | 0 | 1 (2.9)^c^ | 0 | 0 | 3 (6)^c^ | |
| **Cholesterol and free lipid acid alteration** | n/a | n/a | n/a | n/a | n/a | |
| **Skin and subcutaneous tissue disorders** | 1 (2.9) | 5 (14.7) | 1(3) | 0 | 4 (8) | |
| **Eye disorders** | 0 | 0 | 0 | 0 | 0 | |
| **Gastrointestinal disorder** | 1 (2.9) | 1 (2.9) | n/a | n/a | n/a | |
| **Nervous system disorders** | 1 (2.9) | 2 (5.9) | 2 (5) | 5 (13) | 0 | |
| **Psychiatric disorders** | 1 (2.9) | 1 (2.9) | n/a | n/a | n/a | |
| **Renal Disorders** | 0 | 1 (2.9) | n/a | n/a | n/a | |
| **Cardiac and vascular**  **Disorders** | n/a | n/a | n/a | n/a | n/a | |
| **Allergy** | 0 | 0 | n/a | n/a | n/a | |
| **Cancer** | n/a | n/a | n/a | n/a | n/a | |
| **Serious adverse**  **Events** | 0 | 0 | 1 (2) | 0 | 0 | |
| **Deaths** | 0 | 0 | 0 | 0 | 0 | |

^a^Hämoglobin

^b^Neutrophiles

^c^Lymphocytes

^d^Platelets

^e^sum of enrolled patients from both OPT pivotal 1 and OPT pivotal 1 taking 5mg Tofacitinib daily

^e^sum of enrolled patients from both OPT pivotal 1 and OPT pivotal 1 taking 10mg Tofacitinib daily

**Supplementary table 2**: Selected adverse events in a phase II trial in systemic lupus erythematosus

| **Disease type** | Systemic Lupus Erythematosus | | |
| --- | --- | --- | --- |
| **Reference** | Wallace et al. 2018 | | |
| **Study Type** | Phase II  (NCT02708095) | | |
| **Drug** | Baricitinib 2mg | Baricitinib 4mg | Placebo |
| **N^○^ of**  **patients** | 105 | 104 | 105 |
| **Observational period** | 24 weeks | | |
| **Infections of the upper respiratory tract** | 17 (16.2) | 18 (17.3) | 4 (3.8) |
| **Nasopharyngitis** | 6 (5.7) | 5 (4.8) | 3 (2.9) |
| **Other infections** | 11 (10.5) | 9 (8.7) | 11 (10.5) |
| **Fungal**  **infections** | 0 | 0 | 0 |
| **Skin infections** | 0 | 0 | 0 |
| **Herpes virus**  **reactivation** | 0 | 0 | 0 |
| **Liver / renal paramater increase** | 6 (5.8) | 16 (13.5) | 5 (4.8) |
| **Blood Count alteration** | 26 (24.8)^a^  16 (15.3)^b^  25 (23.8)^c^  5 (4.8)^d^ | 33 (31.7)^a^  29 (27.9)^b^  35 (33.7)^c^  6 (5.8)^d^ | 25 (20)^a^  17 (16.2)^b^  40 (38.1)^c^  1 (1)^d^ |
| **Cholesterol and free lipid acid alteration** | 9 (11.7) | 16 (20.7) | 6 (7.5) |
| **Skin and subcutaneous tissue disorders** | 0 | 0 | 1 (1) |
| **Eye disorders** | n/a | n/a | n/a |
| **Gastrointestinal disorder** | 1 (1) | 3 (2.9) | 1 (1) |
| **Nervous system disorders** | 8 (7.6) | 3 (2.9) | 3 (2.9) |
| **Psychiatric disorders** | 2 (1.9) | 2 (1.9) | 0 |
| **Renal Disorders** | 0 | 1 (1) | 0 |
| **Cardiac and vascular**  **Disorders** | 2 (1.9) | 1 (1) | 0 |
| **Allergy** | n/a | n/a | n/a |
| **Cancer** | 0 | 0 | 0 |
| **Serious adverse**  **Events** | 11 (10) | 10 (10) | 5(5) |
| **Deaths** | 0 | 0 | 0 |

**Supplementary Table 3**: Selected adverse events in phase II and III clinical trials for psoriasis

| **Disease**  **type** | PSO | | | | | | | | | | | | | | | | | | | | | | | | | |
| --- | --- | --- | --- | --- | --- | --- | --- | --- | --- | --- | --- | --- | --- | --- | --- | --- | --- | --- | --- | --- | --- | --- | --- | --- | --- | --- |
| **Ref** | Zhang et al. 2017 [] | | | Papp et al., 2015 [121] | | | Bissonnette et al., 2014 [122] | | Bachelez et al.,  2015 [123] | | | | Gladman et al.,  2017 [124] | | | Papp et al., 2016  [128] | | | | | Papp et al., 2018 [129] | | | | | |
| **Study**  **Type** | Phase III  (NCT01815424) | | | Phase III  (NCT012776639)  (NCT(01309737) | | | Phase III  (NCT01186744) | | Phase III  (NCT01186744) | | | | Phase III  (NCT01882439) | | | Phase IIb  (NCT01490632) | | | | | Phase II  (NCT02931838) | | | | | |
| **Drug** | Tofacitinib  5mg BID | Tofacitinib  10mg BID | Placebo | Tofacitinib  5mg BID | Tofacitinib  10mg BID | Placebo | Tofacitinib  5mg BID | Tofacitinib  10mg BID  335 | Tofacitinib  5mg BID | Tofacitinib  10mg BID | Etanercept 50mg twice weekly | Placebo | Tofacitinib  5mg BID | Tofacitinib  10mg BID | Placebo | Baricitinib 2mg | Baricitinib 4mg | Baricitinib 8mg | Baricitinib 10mg | Placebo | BMS  986165  3mg every 2days | BMS  986165  3mg pro die | BMS  986165  3mg BID | BMS  986165  6mg BID | BMS  986165  12mg daily | Placebo |
| **N^○^ of**  **patients** | 88 | 90 | 88 | 745^e^ | 741 | 370 | 336 | 338 | 329 | 330 | 335 | 107 | 132 | 132 | 131 | 32 | 72 | 64 | 69 | 34 | 44 | 44 | 45 | 45 | 44 | 45 |
| **Observational period** | 52 weeks | | | 16 weeks | | | 24weeks | | 12 weeks | | | | 3 months | | | 12 weeks | | | | | 12 weeks | | | | | |
| **Infections of the upper respiratory tract** | 18 (20.5) | 11 (12.2) | 3  (3.4) | 35 (9.4) | 45  (12.2) | 11  (5.9) | 18 (5.4) | 20 (6.0) | 6 (1.8) | 4 (1.2) | 7 (2.1) | 0 | 10 | 6 | 6 | 1 (3.1) | 0 | 1 (1.6) | 2 (2.9) | 1 (2.9) | 1 (2) | 3 (7) | 1 (2) | 4 (9) | 1 (2) | 0 |
| **Nasopharyngitis** | 12 (13.6) | 11 (12.2) | 3  (3.4) | 53 (13.9) | 61  (16.5) | 31  (16.9) | 27 (8.2) | 29 (8.7) | 21 (6.4) | 30 (9.1) | 25 (7.5) | 10 (9.3) | 7 | 10 | 7 | 1 (3.1) | 2 (2.8) | 6 (9.4) | 6 (8.7) | 9 (26.5) | 1 (2) | 4 (9) | 5 (11) | 7 (16) | 2 (5) | 2 (4) |
| **Other infections** | 2 (2.3) | 2 (2.2) | 0 | 1 (0.3) | 4 (1.1) | 0 | 14 (4.2) | 30 (8.8) | 29 (8.1) | 37 (11.1) | 35 (10.5) | 12 (10.6) | 3 | 6 | 7 | 5 (15.5) | 8 (11.2) | 11 (17.6) | 7 (11) | 4 (11..6) | n/a | n/a | n/a | n/a | n/a | n/a |
| **Fungal**  **infections** | n/a | n/a | n/a | n/a | n/a | n/a | n/a | n/a | 6 (1.8) | 1 (0.3) | 4 (1.2) | 1 (0.9) | n/a | n/a | n/a | 1 (3.1) | 0 | 0 | 0 | 0 | 0 | 0 | 0 | 0 | 0 | 0 |
| **Skin infections** | 0 | 5 (5.6) | 1 (1.1) | 1 (0.3) | 0 | 0 | 3 (0.9) | 7 (2.1) | 1 (0.3) | 2 (0.6) | 3 (0.9) | 3 (2.8) | n/a | n/a | n/a | 1 (3.1) | 2  (2.8) | 0 | 0 | 1 (2.9) | 0 | 0 | 0 | 0 | 0 | 0 |
| **Herpes virus**  **reactivation** | 3 (3.4) | 5 (5.6) | 0 | 14 (3.8) | 16 (4.4) | 0 | 0 | 4 (1.2) | 3 (0.9) | 11 (2.1) | 9 (2.4) | 0 | 1 (1) | 1 (1) | 0 | 0 | 0 | 1 (1.6) | 1 (1.4) | 1 (2.9) | 0 | 0 | 0 | 0 | 0 | 0 |
| **Liver / renal paramater increase** | 13 (15.7) | 25(27.9) | 1 (1.1) | 16  (4.5) | 23 (6.3) | 2 (1) | n/a | n/a | n/a | n/a | n/a | n/a | 68  (52) | 71  (54) | 33 (26) | n/a | n/a | n/a | n/a | n/a | 0 | 0 | 0 | 0 | 0 | 0 |
| **Blood Count alteration** | 0^a^  0^b^  0^c^  0^d^ | 1(1.1)^a^  0^b^  0^c^  2 (2.2)^d^ | 0^a^  0^b^  0^c^  0^d^ | 2 (0.6)^a^  0^b^  1 (0.3)^c^  n/a^d^ | 2 (0.6)^a^  1 (0.3)^b^  4(1.1)^c^  n/a^d^ | 0^a^  0^b^  0^c^  0^d^ | n/a | n/a | n/a | n/a | n/a | n/a | 0 | 2  (2) | 1  (0.8) | 0^a^  0^b^  0^c^  n/a^d^ | 1 (1.4)^a^  0^b^  0^c^  n/a^d^ | 1 (1.6)^a^  0^b^  3 (4.7)^c^  n/a^d^ | 2 (2.9)^a^  3 (4.3)^b^  0^c^  n/a^d^ | 0^a^  0^b^  0^c^  0^d^ | n/a^a,b,c,d^ | n/a^a,b,c,d^ | n/a^a,b,c,d^ | n/a^a,b,c,d^ | n/a^a,b,c,d^ | n/a^a,b,c,d^ |
| **Cholesterol and free lipid acid alteration** | 33 (37.5) | 38(42.3) | 13 (14.8) | 29 (7.7) | 29  (7.7) | 5  (2.6) | n/a | n/a | 21 (6.6) | 29 (8.7) | 8 (2.4) | 3 (2.8) | n/a | n/a | n/a | n/a | n/a | n/a | n/a | n/a | 0 | 0 | 0 | 0 | 0 | 0 |
| **Skin and subcutaneous tissue disorders** | 1 (1.1) | 5 (5.6) | 5  (5.7) | 7 (2) | 5 (1.3) | 17  (9.3) | n/a | n/a | n/a | n/a | n/a | n/a | n/a | n/a | n/a | n/a | n/a | n/a | n/a | n/a | 2 (4) | 4 (9) | 3 (6) | 5 (11) | 6 (14) | 4 (8) |
| **Eye disorders** | n/a | n/a | n/a | n/a | n/a | n/a | n/a | n/a | n/a | n/a | n/a | n/a | n/a | n/a | n/a | n/a | n/a | n/a | n/a | n/a | n/a | n/a | n/a | n/a | n/a | n/a |
| **Gastrointestinal disorder** | 6 (6.8) | 7 (7.8) | 0 | n/a | n/a | n/a | 29 (8.7) | 36 (10.8) | 29  (8.8) | 29  (8.8) | 29  (8.7) | 10  (9.3) | 6 (4.5) | 5 (3.8) | 1 (0.8) | n/a | n/a | n/a | n/a | n/a | 5 (11) | 1 (2) | 3 (6) | 3 (6) | 6 (14) | 4 (9) |
| **Nervous system disorders** | n/a | n/a | n/a | 41 (11.1) | 42  (11.4) | 11  (5.9) | n/a | n/a | n/a | n/a | n/a | n/a | 5 (3.8) | 10 (7.6) | 7 (5.3) | n/a | n/a | n/a | n/a | n/a | 4 (9) | 4 (9) | 3 (7) | 3 (7) | 2 (5) | 2 (4) |
| **Psychiatric disorders** | n/a | n/a | n/a | n/a | n/a | n/a | n/a | n/a | n/a | n/a | n/a | n/a | n/a | n/a | n/a | n/a | n/a | n/a | n/a | n/a | n/a | n/a | n/a | n/a | n/a | n/a |
| **Renal Disorders** | 4 (4.5) | 5 (5.6) | 1  (1.1) | n/a | n/a | n/a | n/a | n/a | n/a | n/a | n/a | n/a | n/a | n/a | n/a | n/a | n/a | n/a | n/a | n/a | n/a | n/a | n/a | n/a | n/a | v |
| **Cardiac and vascular**  **Disorders** | n/a | n/a | n/a | 2 (0.5) | 1 (0.3) | 0 | 1 | 0 | 1 (0.3) | 0 | 0 | 0 | 4 (1.5) | 4 (1.5) | 2 (1.5) | n/a | n/a | n/a | n/a | n/a | 0 | 0 | 0 | 0 | 0 | 0 |
| **Allergy** | n/a | n/a | n/a | n/a | n/a | n/a | n/a | n/a | n/a | n/a | n/a | n/a | n/a | n/a | n/a | n/a | n/a | n/a | n/a | n/a | n/a | n/a | n/a | n/a | n/a | n/a |
| **Cancer** | 1  (1.1) | 1  (1.1) | 0 | 2 (0.5) | 2 (0.5) | 0 | 0 | 3 (1.9)  0 | 1 (0.3) | 0 | 1 (0.3) | 0 | 0 | 0 | 0 | 0 | 1 (1.4) | 0 | 0 | 0 | 0 | 1 (2) | 0 | 0 | 0 | 0 |
| **Serious adverse**  **Events** | 4 (4.5) | 2 (2.2) | 0 | 19 (5.1) | 15 (4.1) | 6  (3.8) | 11 (3.3) | 22 (6.6) | 7 (2) | 5 (2) | 7 (2) | 2 (2) | 3 (2) | 1 (1) | 3 (2) | 1 (3.1) | 1 (1.4) | 1 (1.6) | 1 (1.6) | 1 (2.9) | 1 (2) | 1 (2) | 1 (2) | 0 | 0 | 1 (2) |
| **Deaths** | 1 (1.1) | 0 | 0 | 2 (0.6) | 0 | 0 | 1 (0.3) | 0 | 0 | 0 | 0 | 0 | 0 | 0 | 0 | 0 | 1 (1.4) | 0 | 0 | 0 | 0 | 0 | 0 | 0 | 0 | 0 |
